# Estimation of the Comprehensive High Photosynthetic-Efficiency Phenotypic Index in Winter Wheat Based on UAV Multimodal Remote Sensing Data

**DOI:** 10.3390/plants15172674

**Published:** 2026-08-31

**Authors:** Ning Yang, Dayong Cui, Songming Lin, Changliang Du, Liwen Wang, Fei Zhang, Zhu Shi, Bingqian Hou, Junke Zhu

**Affiliations:** 1 School of Life Science, Qilu Normal University, Jinan 250200, China; yangning1228@126.com (N.Y.); cuidayong@qlnu.edu.cn (D.C.);; 2 School of Agricultural and Food Engineering, Shandong University of Technology, Zibo 255000, China

**Keywords:** UAV, winter wheat, phenotypic monitoring, photosynthetic efficiency

## Abstract

Accurate monitoring of photosynthetic phenotypes is fundamental for breeding high photosynthetic-efficiency wheat cultivars, and UAV remote sensing provides an effective approach for their large-scale identification. However, single photosynthetic parameters are limited in comprehensively evaluating crop photosynthetic efficiency. This study integrated multiple photosynthetic phenotypic parameters using principal component analysis (PCA) and the CRITIC objective weighting method (PCA-CRITIC) to construct a Comprehensive High Photosynthetic-Efficiency Phenotypic Index (CHPPI) for winter wheat. Concurrently, multispectral vegetation indices (MSVI), RGB vegetation indices (RGBVI), texture features (TF), and their combination, Comprehensive Multispectral-Visible-Texture Features (CMVTF), were extracted from UAV multimodal remote sensing data. Based on these features, four feature selection methods, namely PCC, VIP, SPA, and UVE, were evaluated in combination with five machine learning algorithms, including KNN, DT, SVR, RF, and XGBoost, to precisely estimate the CHPPI across key growth stages. The results demonstrated that the CHPPI exhibited significant cultivar variations across growth stages. Cultivars SH06144 and Liangxing19 consistently maintained high photosynthetic efficiency levels. The fused feature set CMVTF derived from multimodal remote sensing data outperformed individual feature categories, and the feature subset selected by the UVE method most significantly enhanced model accuracy. Regarding algorithms, the ensemble-based XGBoost and RF models markedly surpassed traditional machine learning algorithms. Specifically, the CMVTF-UVE-XGBoost model achieved the optimal comprehensive performance, yielding an R^2^ of 0.862, an RMSE of 0.032, and an MAE of 0.027 on the testing set, with its spatial distribution estimations highly consistent with measured values. This study provides robust technical support for the rapid and large-scale screening of high photosynthetic-efficiency wheat breeding materials.

## 1. Introduction

Winter wheat represents one of the major food crops worldwide. Breeding its elite varieties can not only safeguard food security and stabilize grain supply, but can also play a vital role in supporting sustainable agricultural development [1,2]. Meanwhile, crop phenotype is the comprehensive manifestation of appearance morphology, physiological functions, and physicochemical properties under the joint action of genotype and environment [3], and accurate, efficient crop phenotype identification stands as a key bottleneck in modern precision breeding [4,5]. As a type of crop physiological function phenotype, photosynthetic phenotype directly characterizes plant carbon assimilation capacity and environmental adaptability, serving as a critical trait that determines crop dry matter accumulation, yield formation, and stress resistance [6,7]. Therefore, screening superior materials and breeding high-photosynthetic-efficiency varieties from the perspective of photosynthetic phenotyping is considered an important direction for elevating wheat yield potential and improving variety traits [8]. 

High photosynthetic-efficiency wheat typically exhibits stronger carbon assimilation, superior stomatal regulation, and more coordinated photochemical energy conversion [9]. However, in current crop phenotyping and breeding practices, photosynthetic phenotypic indicators—such as net photosynthetic rate, stomatal conductance, and chlorophyll fluorescence—heavily rely on manual, point-by-point measurements using handheld devices. This labor-intensive and time-consuming approach limits sample sizes, failing to meet the demand for high-throughput screening of breeding materials [10,11]. Furthermore, a single indicator can only reflect a localized feature of the photosynthetic physiological process, failing to fully characterize the comprehensive performance of wheat photosynthetic efficiency. Principal component analysis (PCA) can effectively eliminate collinear redundancy among multiple physiological parameters and extract core mutational information through dimensionality reduction [12], while the CRITIC objective weighting method can allocate weights based on the contrast intensity and conflict among indicators, avoiding the subjectivity of traditional manual weighting [13]. Therefore, combining these methods to construct a comprehensive indicator enables a more comprehensive and robust evaluation of the integrated photosynthetic productivity of winter wheat populations than single indicators.

In recent years, unmanned aerial vehicle (UAV) remote sensing technology has provided critical technical support for the high-throughput acquisition of crop phenotypes [14,15]. Compared with UAV hyperspectral remote sensing, UAV RGB and multispectral remote sensing offer significant advantages, including high cost-effectiveness, convenient data processing, and strong sensor stability. Furthermore, the vegetation index constructed from spectral bands can precisely respond to the dynamic changes in crop phenotypes [16,17]. However, in dense canopies with high biomass, utilizing only spectral information to perceive changes in crop physiological status may be limited due to the asymptotic saturation effect of optical sensors [18]. Conversely, the texture feature can reflect the spatial physical properties of the canopy from the dimension of geometric structures, effectively mitigating the spectral saturation phenomenon and improving the estimation accuracy of crop phenotypic parameters [19,20].

In the process of collaborative modeling using multimodal remote sensing features, the original feature variables suffer from severe dimensional redundancy and collinearity issues, which greatly limit the model’s accuracy and generalization capability [21]. Therefore, introducing an efficient feature selection method is critical for modeling. Feature selection can not only effectively identify and extract feature subsets that possess a strong driving capacity toward the target variable, thereby reducing model complexity [22], but more importantly, it can also efficiently suppress model overfitting, enhancing the robustness of model predictions [23]. Regarding modeling algorithms, machine learning algorithms have demonstrated significant advantages in crop phenotype estimation due to their excellent non-linear mapping capabilities [24]. Notably, the application of ensemble learning, by integrating the strengths of multiple weak learners, can provide stronger robustness while maintaining high estimation accuracy [25,26]. Consequently, utilizing a modeling strategy that combines feature selection methods with machine learning algorithms to improve the estimation accuracy of winter wheat CHPPI aims to achieve precise monitoring of crop photosynthetic efficiency levels, thereby effectively screening high photosynthetic-efficiency winter wheat varieties.

Therefore, this study combined the PCA and CRITIC method to construct the Comprehensive High Photosynthetic-Efficiency Phenotypic Index (CHPPI) for the quantitative characterization of winter wheat photosynthetic phenotypes, and comparatively analyzed the dynamic regularities of CHPPI across different varieties and growth stages;

The RGB vegetation index (RGBVI), multispectral vegetation index (MSVI), and texture feature (TF) extracted from UAV spectral images, as well as their combinations, were utilized to estimate the CHPPI, while the performance of machine learning algorithms under various feature selection strategies was evaluated; based on the optimal estimation model, the spatial distribution maps of CHPPI were generated. This study aims to establish a method for estimating the photosynthetic phenotype based on UAV multimodal remote sensing data, thereby providing technical support for the rapid and non-destructive screening of high photosynthetic-efficiency winter wheat varieties.

## 2. Results

### 2.1. Analysis of Varietal Differences in Winter Wheat CHPPI

**Figure 1:**
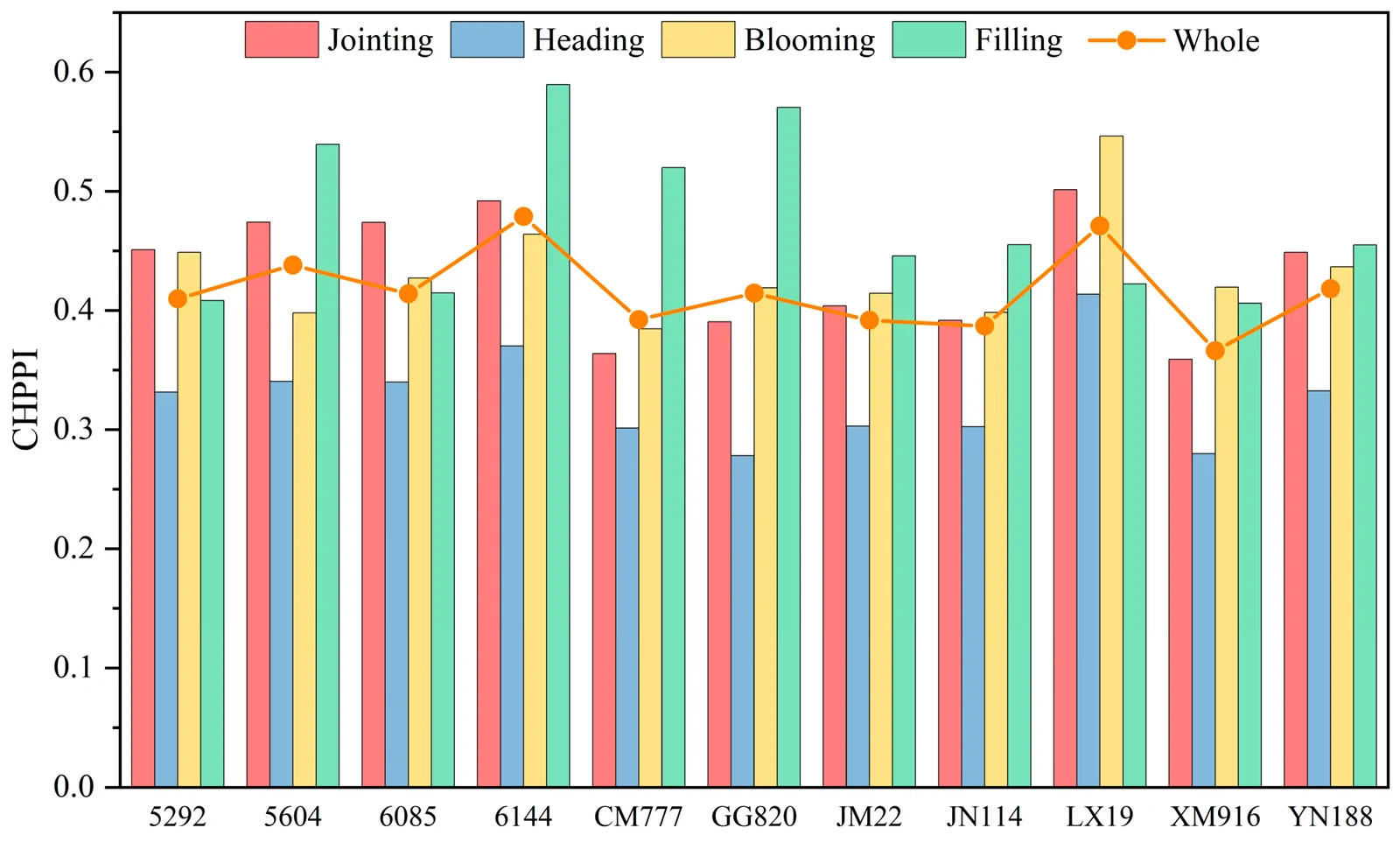
CHPPI values of different cultivars across growth stages.

Quantitative evaluation of the CHPPI values across 11 winter wheat varieties at different growth stages revealed significant growth-stage fluctuations and varietal variations (Figure 1). Regarding temporal dynamics, most varieties exhibited high CHPPI values at the jointing stage, followed by a general, phased decline during the heading stage. However, upon entering the flowering stage, the CHPPI values displayed a marked upward trend, with most varieties reaching or approaching their secondary peaks of the entire growth period. Subsequently, at the grain-filling stage, distinct and diversified trends emerged depending on specific varietal characteristics.

In terms of varietal performance, ‘6144’ and ‘LX19’ exhibited the highest photosynthetic efficiency throughout the growth period, with CHPPI values of 0.479 and 0.471, respectively. Notably, ‘6144’ maintained a CHPPI value of 0.589 during the grain-filling stage, far exceeding other varieties and indicating an exceptional photosynthetic maintenance capability during the late growth stage. In contrast, ‘XM916’ and ‘JN114’ displayed the lowest photosynthetic efficiency, with CHPPI values of 0.366 and 0.387, respectively. This distinct varietal gradient validates the sensitivity of CHPPI in evaluating wheat photosynthetic efficiency levels, effectively differentiating variations in photosynthetic productivity among varieties.

### 2.2. Correlation Analysis Between CHPPI and Feature Variables

**Figure 2:**
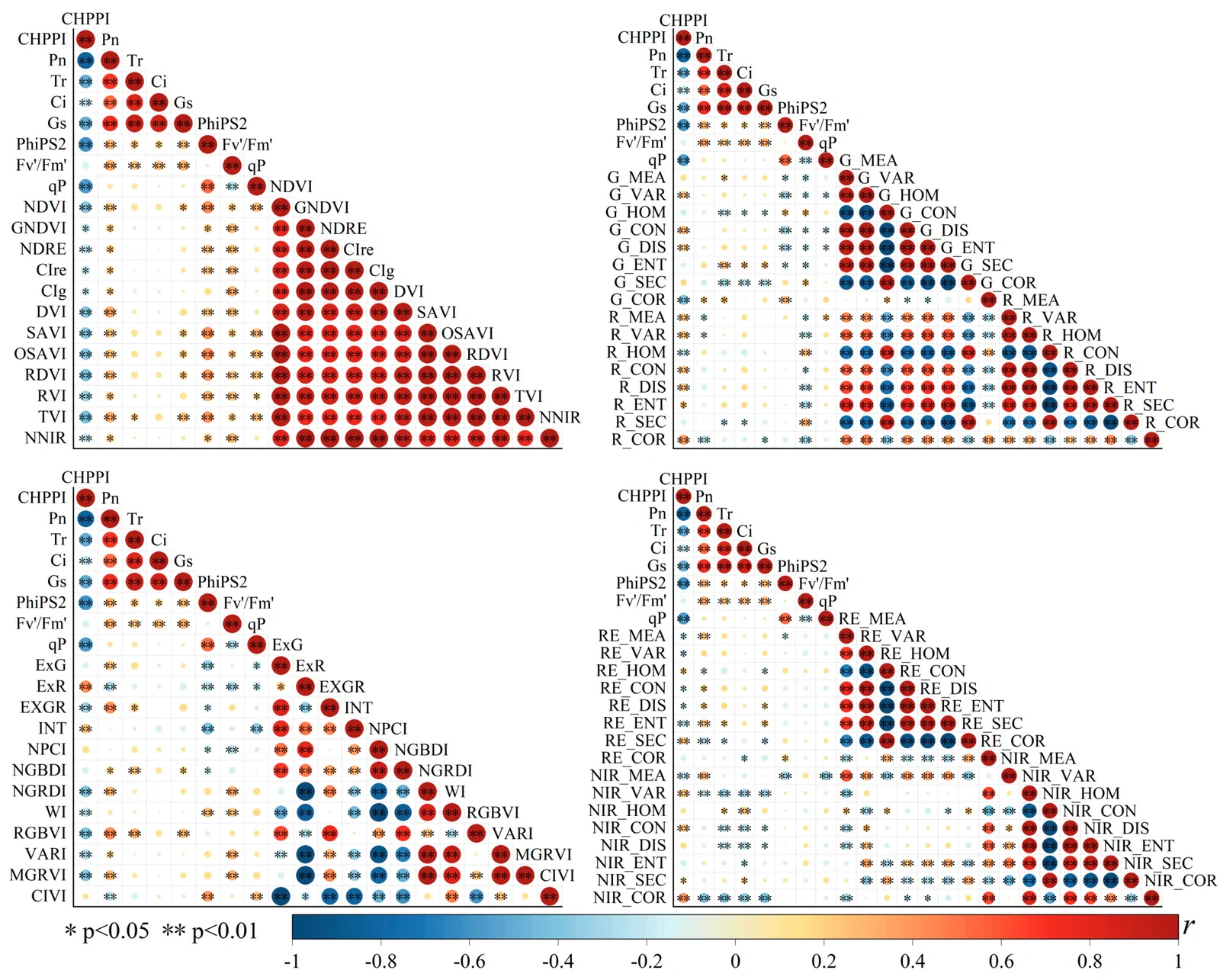
Correlations between CHPPI and different feature variables.

The correlations between the CHPPI and different types of feature variables are illustrated in Figure 2. The correlations between the CHPPI and the feature variables were generally higher than those observed for single photosynthetic parameters, such as Pn, Tr, Ci. Among the RGBVIs, ExR (r = 0.429) and RGBVI (r = −0.413) exhibited the highest correlation coefficients with the CHPPI, both achieving significance at the 0.01 level. Within the MSVIs, TVI and RDVI showed highly significant negative correlations with the CHPPI (*p* < 0.01), with *r* values of −0.453 and −0.42, respectively. Furthermore, among the TFs, G_COR and NIR_COR also displayed higher correlations with the CHPPI than with single photosynthetic parameters, yielding *r* values of −0.407 and 0.420, respectively (*p* < 0.01). These findings strongly demonstrate that by integrating multidimensional photosynthetic information, the CHPPI provides a more robust representation of high-light-efficiency levels in winter wheat, effectively characterizing its growth status under complex field environments.

### 2.3. Effects of Different Feature Variables on CHPPI Estimation

When constructing machine learning models, input variable selection directly determines estimation performance. This study compared the performance of MSVI, RGBVI, TF, and CMVTF, as model inputs (Table 1). Results indicated that among single feature types, MSVI generally outperformed RGBVI and TF. Furthermore, the multi-source CMVTF dataset demonstrated significant superiority across all algorithms, far exceeding single-source models. This confirms that the canopy spatial structure information provided by texture features effectively complements spectral data in physiological monitoring. Additionally, the ensemble learning algorithms (XGBoost and RF) constructed more accurate CHPPI models than SVR, DT, and KNN, achieving testing R^2^ values of 0.725 and 0.703, and reducing RMSE to 0.045 and 0.038, respectively. This demonstrates that ensemble learning possesses superior fitting capabilities when handling multimodal feature datasets, thereby enhancing CHPPI estimation reliability.

### 2.4. Feature Selection Based on Different Methods

**Figure 3:**
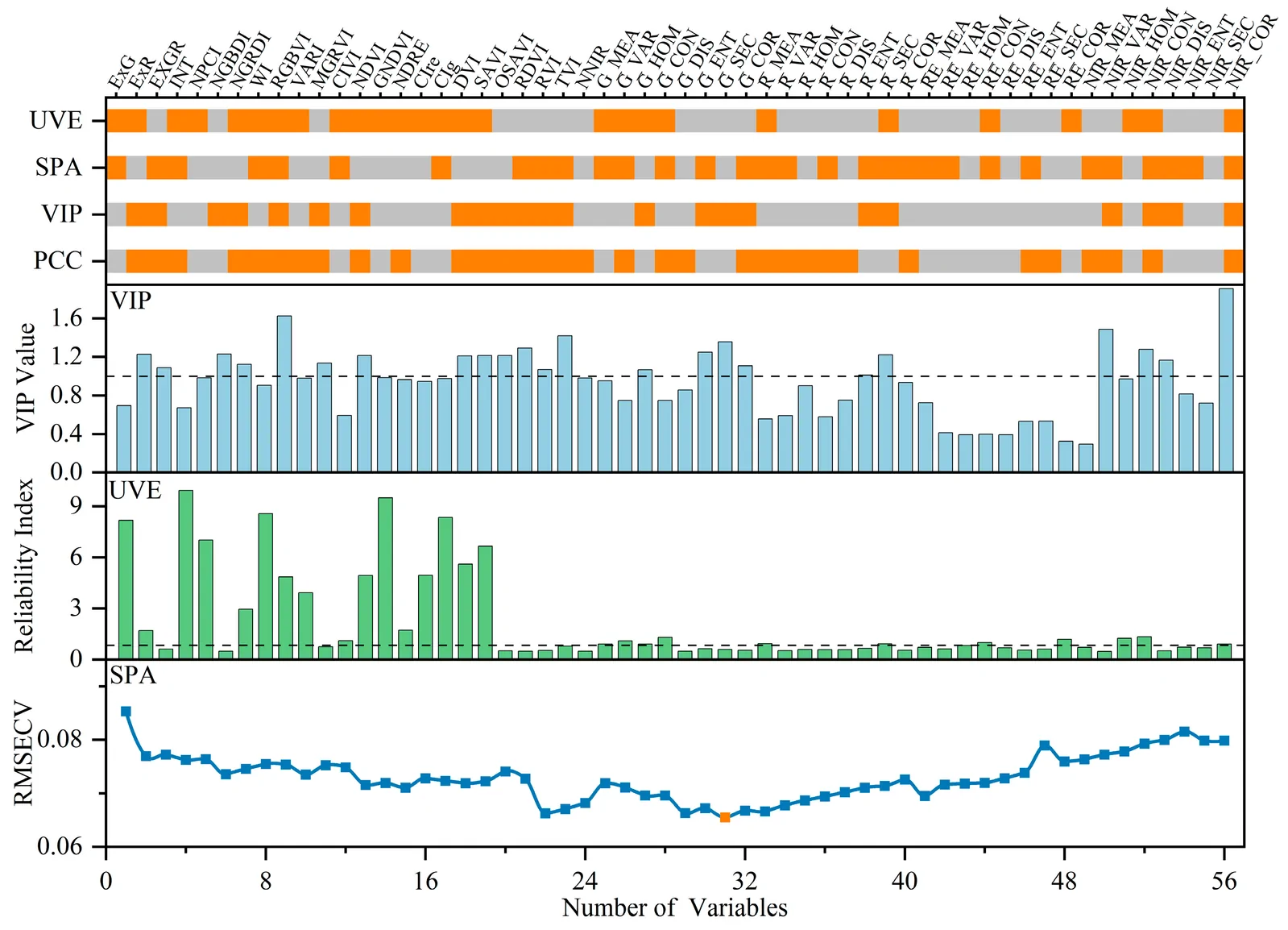
Feature selection based on different feature selection methods.

To eliminate redundancy among the 56 original features and suppress model overfitting, four feature selection methods, including PCC, VIP, SPA, and UVE, were employed for dimensionality reduction (Figure 3). Specifically, the PCC method focused on retaining variables with strong linear relationships, selecting 33 highly significantly correlated features. The VIP method, based on partial least squares projections, screened 23 core features with high explanatory power. Leveraging vector space projection, the SPA minimized collinearity interference to retain 31 mutually complementary features. Lastly, the UVE method evaluated feature stability by introducing random noise, ultimately identifying 27 robust feature variables. These feature selection strategies effectively reduced the number of variables, establishing a foundation for constructing parsimonious and efficient estimation models.

### 2.5. Effects of Feature Selection Methods on Estimation Performance

**Figure 4:**
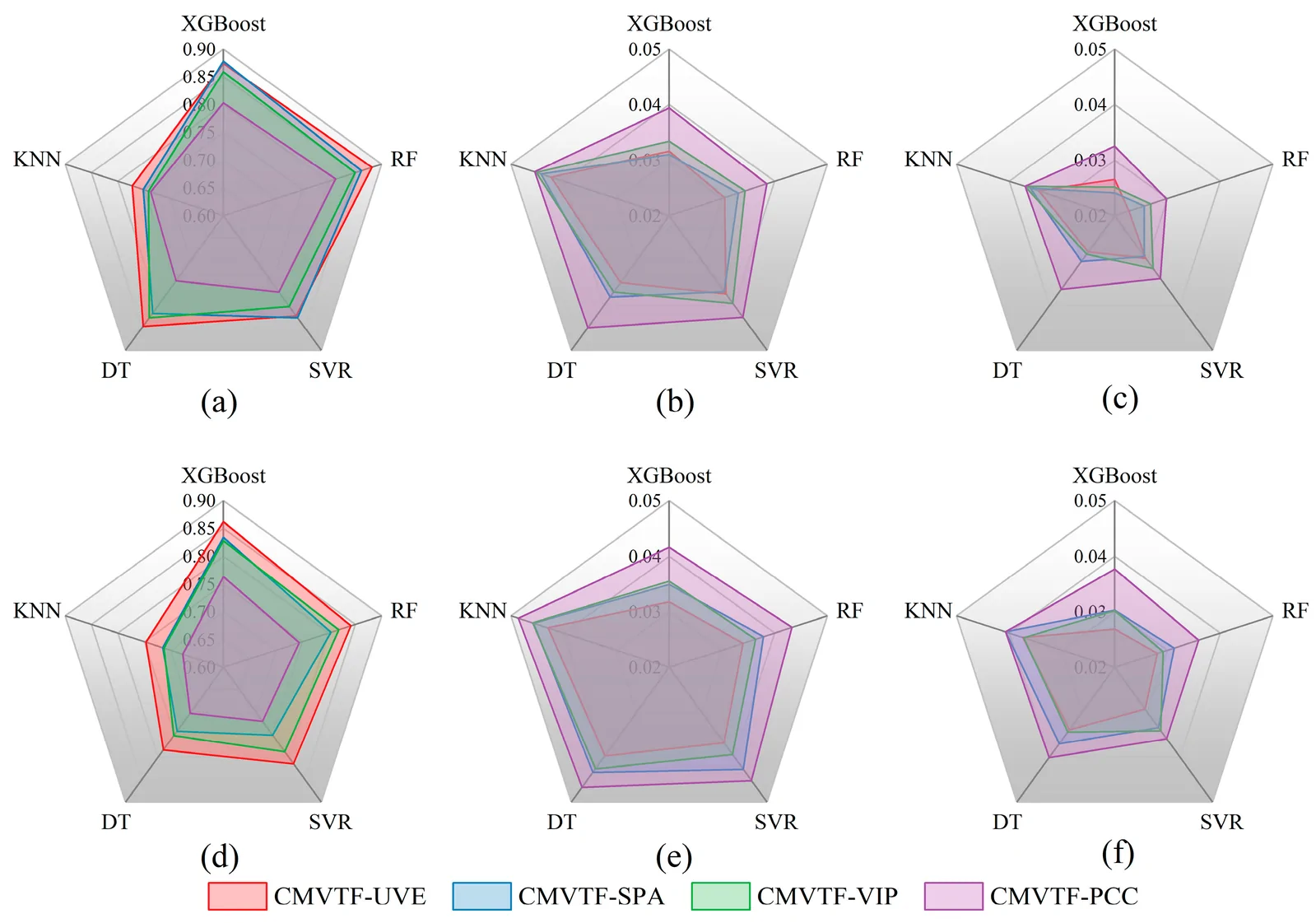
Performance of CHPPI estimation models based on different feature selection methods. Notes: (**a**–**c**) represent the R^2^, RMSE, and MAE of the training dataset, respectively; (**d**–**f**) represent the R^2^, RMSE, and MAE of the testing dataset, respectively.

The feature subsets, which included CMVTF-PCC, CMVTF-VIP, CMVTF-SPA and CMVTF-UVE, selected from the CMVTF dataset by different feature selection methods, were utilized to construct and evaluate the performance of the estimation models as shown in Figure 4. The results indicated that the CHPPI estimation models built after feature selection showed varying degrees of performance improvement compared with those using full feature inputs. Among these methods, the CMVTF-UVE variable combination demonstrated the highest robustness, enabling the XGBoost and RF algorithms to achieve the highest accuracy. Specifically, for the training set, the R^2^, RMSE, and MAE values were 0.874, 0.032, and 0.027 for XGBoost, and 0.882, 0.031, and 0.023 for RF, respectively. For the testing set, these respective metrics reached 0.862, 0.032, and 0.027 for XGBoost, and 0.842, 0.034, and 0.029 for RF. These findings indicate that coupling feature selection methods with ensemble learning algorithms offers a clear advantage, effectively increasing the estimation accuracy of CHPPI.

**Figure 5:**
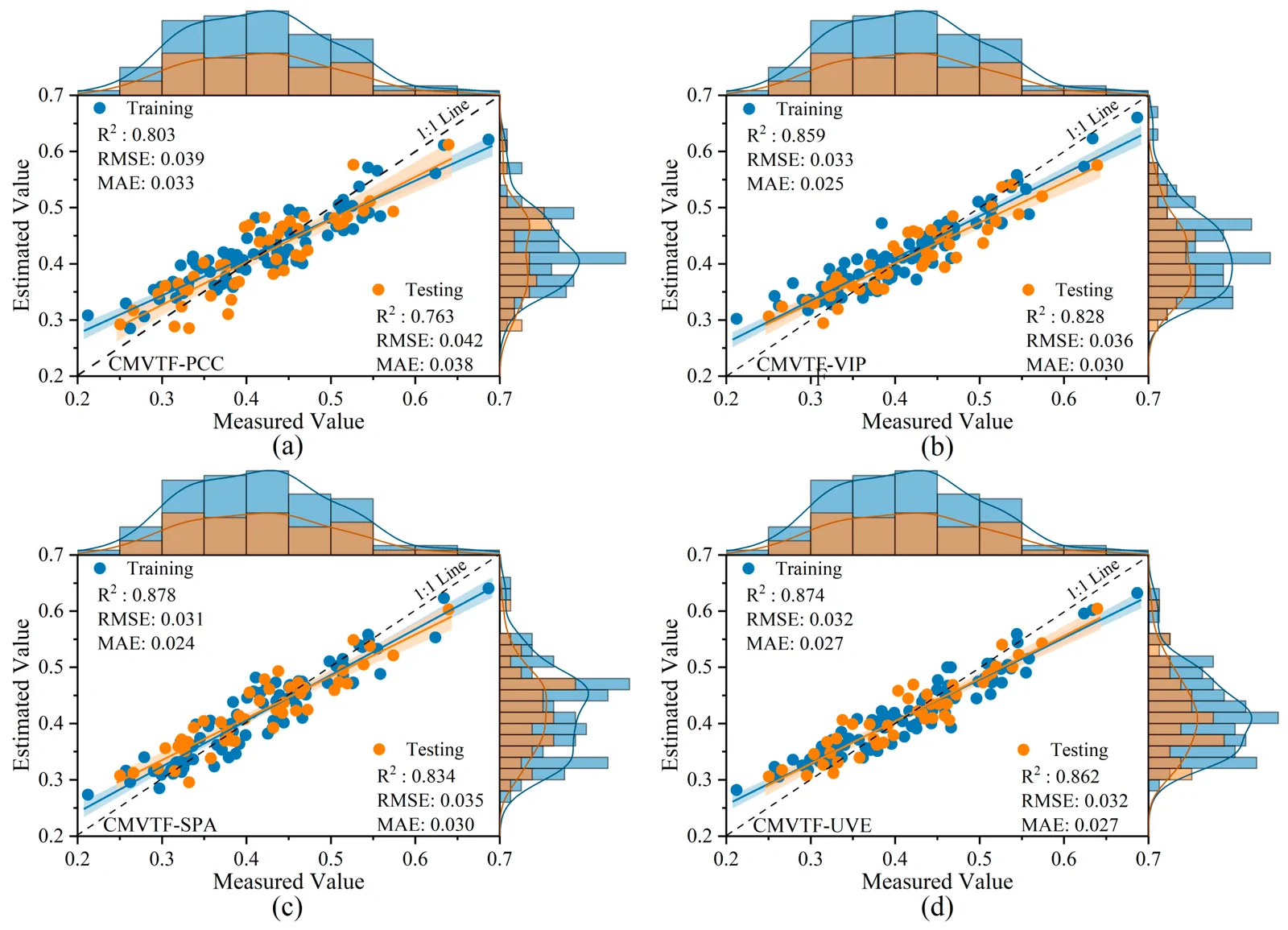
CHPPI estimation results based on the XGBoost model. Notes: (**a**–**d**) represent the CHPPI estimation results obtained for the feature dataset CMVTF selected by PCC, VIP, SPA, and UVE, respectively.

Comprehensive analysis revealed that the CHPPI estimation model constructed using the UVE selected feature subset combined with the XGBoost algorithm, denoted as CMVTF-UVE-XGBoost, achieved the optimal performance (Figure 5d). This model demonstrated high consistency across both the training and testing datasets, with predicted and measured values tightly distributed around the 1:1 line and no significant overfitting. Compared with CMVTF-PCC-XGBoost (Figure 5a), the training and testing R^2^ of CMVTF-UVE-XGBoost increased by 8.84% and 12.98%, while the RMSE and MAE decreased by 17.95% and 23.81% on the training set, and by 18.18% and 28.95% on the testing set, respectively. This further confirms that the UVE method effectively identifies and retains feature subsets highly aligned with crop photosynthetic potential, significantly enhancing model generalization and accuracy by eliminating collinear redundancy.

**Figure 6:**
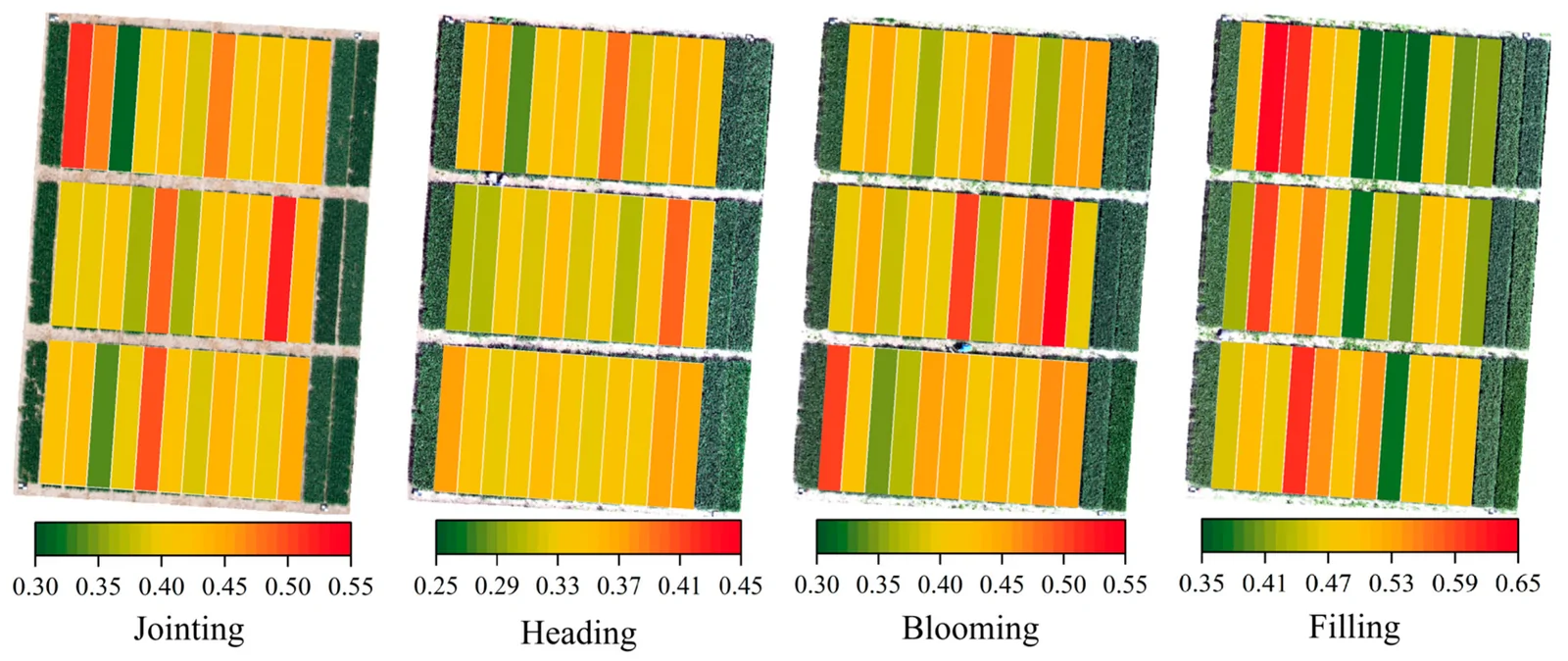
Spatial distribution of wheat CHPPI values across different growth stages.

This study further analyzed the spatial distribution of winter wheat CHPPI based on the CMVTF-UVE-XGBoost model to evaluate its reliability across different growth stages (Figure 6). The results indicated that the distribution of estimated red high-value and green low-value areas of CHPPI was basically consistent with the measured values. This not only fully reflected the dynamic changes in CHPPI during winter wheat growth, but also proved the sensitive capture of the estimation model for the photosynthetic efficiency levels of different winter wheat cultivars. Overall, the model can achieve precise estimation of CHPPI across different growth stages of wheat, providing technical support for the subsequent screening of high photosynthetic efficiency wheat breeding materials.

## 3. Discussion

The CHPPI constructed in this study by coupling PCA with the CRITIC objective weighting method successfully overcomes the limitations of using a single photosynthetic phenotypic parameter to evaluate crop photosynthetic efficiency. The results revealed that the CHPPI of winter wheat peaked during the jointing stage, generally decreased upon entering the heading stage, and subsequently rebounded during the flowering stage. This fluctuating trend is driven by a combination of ambient microclimatic conditions and physiological resource allocation. Environmentally, the heading stage frequently encounters elevated midday temperatures and vapor pressure deficit (VPD), inducing evaporative demand that triggers protective stomatal closure to prevent water loss [27]. This stomatal limitation restricts Ci influx, downregulating Pn [28]. Concurrently, excessive thermal load and photon flux induce transient photoinhibition of open PSII reaction centers, manifested as diminished PhiPS2 and F’/Fm [29]. Physiologically, the vegetative to reproductive transition at heading induces a major source-sink realignment, diverting metabolic energy and nitrogenous compounds toward spike formation and temporarily lowering leaf photosynthetic capacity [30,31]. Conversely, the resurgence of CHPPI values at the flowering stage marks a profound coordination within the photosynthetic structure and function of the plants [32]. Regarding cultivar variations, SH06144 and Liangxing19 exhibited remarkable photosynthetic robustness. Notably, the CHPPI of cultivar SH06144 remained at a high level even during the late growth stage, indicating its superior capacity to sustain elevated photosynthetic activity compared with other cultivars [33]. Such prominent discrepancies in photosynthetic productivity among different cultivars further validate the substantial application potential of CHPPI in evaluating high photosynthetic efficiency breeding materials.

The fusion of multi-source remote sensing features and effective feature selection strategies is critical for the precise estimation of crop phenotypes [34,35]. By integrating MSVI, RGBVI, and TF, this study significantly improved CHPPI estimation accuracy (Table 1). MSVI frequently exhibits asymptotic saturation during the vigorous growth stages of winter wheat due to dense canopies, which limits model performance [36,37]. Conversely, RGBVI sensitively captures subtle pigment variations within visible bands [38], while texture features reflect the physical attributes of the canopy from a geometric structure dimension, thereby mitigating the accuracy loss caused by spectral saturation [39]. Concurrently, the four feature selection strategies employed in this study, namely PCC, VIP, SPA, and UVE, all successfully extracted effective feature variable subsets. Specifically, the UVE method eliminated redundant variables that weakly respond to photosynthetic physiological status and alleviated multi-collinearity among features [40,41], thereby significantly enhancing the precision of the CHPPI estimation model (Figure 4).

In this study, machine learning algorithms mitigated multi-collinearity among remote sensing features and captured their complex non-linear relationships with CHPPI [42]. Specifically, ensemble learning models, represented by XGBoost and RF, outperformed SVR, DT, and KNN in estimation accuracy. This superiority is primarily because XGBoost iteratively minimizes residual loss via a forward stagewise additive strategy, demonstrating powerful capabilities in capturing complex non-linear feature representations [43,44]. Meanwhile, RF reduces model variance and significantly enhances robustness against noise in remote sensing data through its bagging strategy and randomized feature selection [45,46]. Notably, the CMVTF-UVE-XGBoost model achieved the highest CHPPI estimation accuracy on the testing set (R^2^ = 0.862, RMSE = 0.032 and MAE = 0.027) (Figure 5). This indicates that integrating feature selection strategies with ensemble learning algorithms drastically enhances the non-linear fitting and anti-overfitting capabilities of the model [47,48], thereby ensuring highly accurate estimations of crop photosynthetic phenotypes. Furthermore, compared with expensive hyperspectral equipment, this study achieved satisfactory CHPPI estimation results using only visible and multispectral data, substantially reducing costs for large-scale field applications [17,49].

In conclusion, the technical framework combining feature fusion, feature selection, and machine learning can accurately estimate the CHPPI of winter wheat and effectively distinguish cultivar-level photosynthetic potential, offering a rapid pathway for high-photosynthetic-efficiency germplasm screening. Although this study achieved satisfactory CHPPI estimation results, future efforts should focus on integrating deep learning architectures [50] and crop growth models [51] to enhance both the transferability and mechanistic understanding of photosynthetic phenotyping. In addition, while sample-level partitioning across pooled growth stages was used here to build a universal CHPPI model, implementing a leave-one-growth-stage-out cross-validation scheme will be crucial in future studies to rigorously evaluate model generalization across dynamic canopy development.

## 4. Materials and Methods

### 4.1. Study Area and Experimental Design

**Figure 7:**
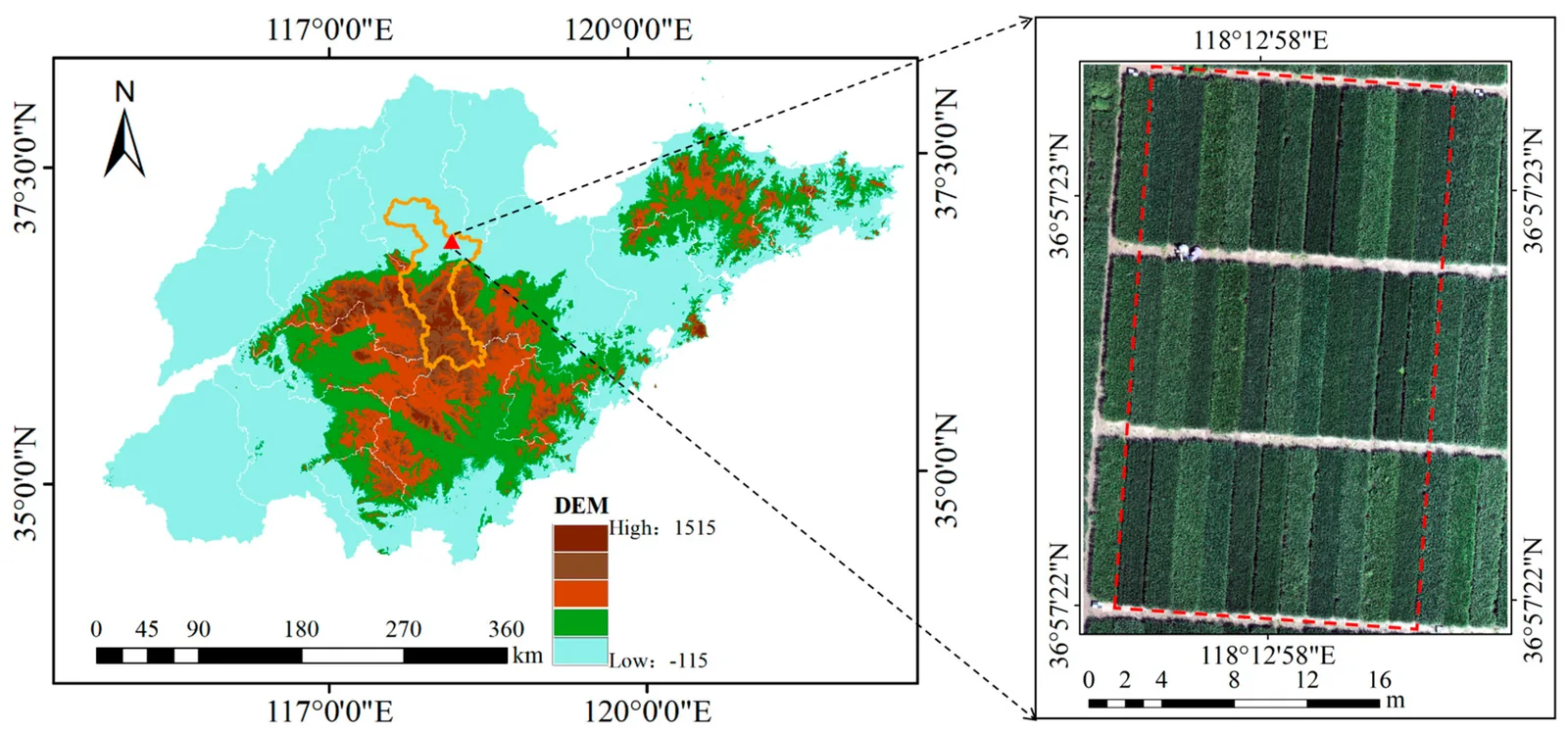
Location of the study area and test plots.

The study area is located at the Ecological Unmanned Farm of Zibo Hefeng Seed Industry Technology Co., Ltd., in Zibo City, Shandong Province (36°57′ N, 118°13′ E) (Figure 7). The experimental site sits at an elevation of approximately 21 m, with annual average sunshine duration ranging from 2100 to 2500 h and a frost-free period spanning 180 to 220 days. Situated in the central region of Shandong Province, this site represents a transition zone between the North China Plain and the central Shandong mountains. It is characterized by a temperate continental monsoon climate, featuring a multi-year average annual precipitation of approximately 650 mm and an annual mean temperature of about 13.2 °C. The climate presents hot, rainy summers with concurrent heat and precipitation, contrastingly paired with cold, dry winters with minimal rain and snow. The predominant soil type within the experimental plots is clay loam, exhibiting a pH value of approximately 7.8, a soil bulk density of around 1.38 g/cm, a field capacity of approximately 23%, and a wilting coefficient of roughly 9.2%.

**Figure 8:**
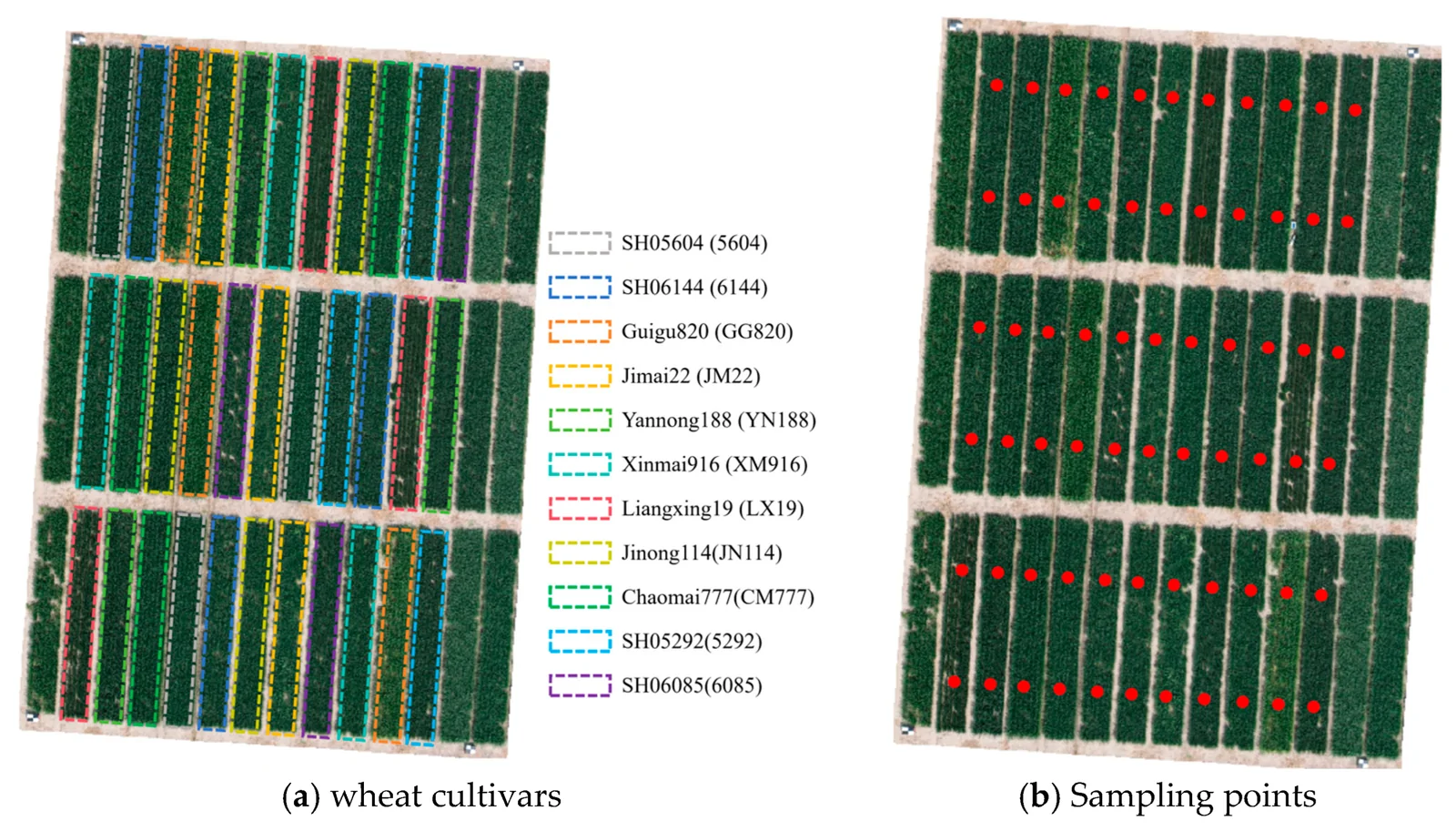
Field distribution of wheat cultivars and sampling points.

Eleven wheat lines were investigated in this study, comprising seven widely cultivated varieties and four independently developed lines currently awaiting official approval. A randomized complete block design with three replications was employed (Figure 8a), resulting in a total of 33 plots. Each plot covered an area of 15 m^2^, with a 0.5 m buffer zone between adjacent plots. Sowing was performed on 13 October 2023, with a row spacing of 30 cm. Basal fertilizer consisted of a compound fertilizer applied at a rate of 450 kg/ha. Supplementary nitrogen, in the form of 46% pure urea, was top-dressed during the tillering stage (150 kg/ha) and from the rising to jointing stages (300 kg/ha), respectively. Field management operations conformed to local high-yield cultivation standards, including scheduled irrigation, weeding, and integrated pest and disease management. The crops were harvested on 11 June 2024, with a total growth duration of approximately 240 days. Field data collection was executed across four critical growth stages: jointing, heading, flowering, and grain filling. Within each plot, two specific locations were marked as sampling points, and their distribution is illustrated in Figure 8b.

### 4.2. Field Data Acquisition and Processing

#### 4.2.1. Acquisition of Photosynthetic Phenotype Parameters

**Figure 9:**
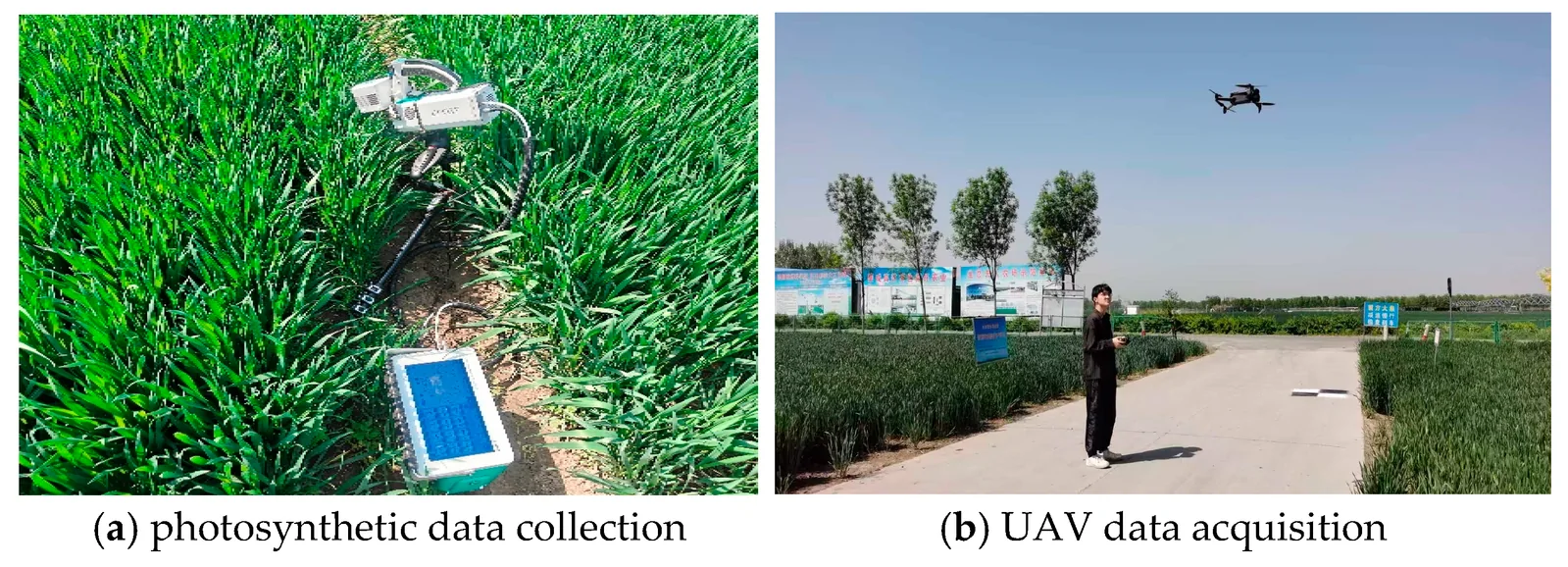
Experimental data acquisition.

Seven photosynthetic phenotypic parameters were monitored using an LI-6800 portable photosynthesis system (LI-COR, Lincoln, NE, USA) (Figure 9). These parameters included net photosynthetic rate (Pn), transpiration rate (Tr), intercellular CO_2_ concentration (Ci), stomatal conductance (Gs), photochemical quantum efficiency (PhiPS2), efficiency of excitation energy capture by open PSII reaction centers (F’/Fm), and the quenching coefficient (qP). Data collection was conducted across four critical growth stages: jointing (April 6), heading (April 24), flowering (May 2), and grain filling (May 11), specifically between 10:00 and 12:00 under clear and cloudless weather conditions. During sampling, three uniform main-stem flag leaves were selected as measurements at each of the two sampling points within every plot. A total of six replicate observational datasets were recorded per plot, and their arithmetic mean was calculated as the final measured value of the photosynthetic phenotypic parameters for that plot.

#### 4.2.2. Construction of Comprehensive Index

Evaluating high-photosynthetic-efficiency varieties using a single parameter is highly susceptible to collinearity interference, making it difficult to fully capture the comprehensive photosynthetic potential of crops. Therefore, this study integrated PCA with the CRITIC objective weighting method (PCA-CRITIC) to construct a Comprehensive High Photosynthetic-Efficiency Phenotypic Index (CHPPI). This index aims to provide a scientific framework for the screening and quantitative characterization of high-photosynthetic-efficiency winter wheat varieties.

During the construction of the CHPPI, the measured photosynthetic phenotypic parameters were first standardized (Equation (1)) to eliminate differences in dimensions and magnitudes among parameters, thereby ensuring data comparability. Subsequently, PCA was applied to reduce the dimensionality of the standardized matrix, transforming these complexly correlated photosynthetic phenotypic parameters into mutually orthogonal principal components to eliminate collinear redundancy among them, and the principal component scores for each sample were calculated (Equations (2)–(4)). According to the standard criterion of retaining a cumulative variance contribution rate exceeding 90% [52], the first four principal components were extracted. To facilitate intuitive comparison and comprehensive evaluation, the principal component scores underwent forward normalization (Equation (5)).

(1) $$X_{ij}^{\prime }=\frac{X_{ij}-\mu_{j}}{\sigma_{j}}$$

(2) $$C=\frac{1}{n-1}(X_{ij}^{\prime }{)}^{T}X_{ij}^{\prime }$$

(3) $$Cv_{k}=\lambda_{k}v_{k}$$

(4) $${\mathrm{PC}}_{k}=X^{\prime }v_{k}$$

(5) $${\mathrm{PC}}_{k}^{norm}=\frac{{\mathrm{PC}}_{k}\;-\min({\mathrm{PC}}_{k})}{\max({\mathrm{PC}}_{k})\;-\min({\mathrm{PC}}_{k})}$$

where X’*_ij_* is the standardized value; X*_ij_* represents the original value of the j-th index for the i-th sample; μ*_j_* and σ*_j_* are the mean and standard deviation of the j-th index, respectively; C denotes the covariance matrix; λ*_k_* is the k-th eigenvalue; v*_k_* represents the corresponding eigenvector; PC*_k_* is the score vector of the k-th principal component; and ${\mathrm{PC}}_{k}^{norm}$ represents the forward normalization of the principal component scores.

After obtaining the principal component scores, the CRITIC objective weighting method was employed to determine the contribution of each principal component within the comprehensive evaluation framework. By considering the information capacity of scores and the conflict among components (Equations (6) and (7)), this approach calculates the objective weight for each component (Equation (8). Consequently, it effectively avoids the subjective bias of manual weight assignments typical in traditional methods. Finally, the CHPPI was constructed using a weighted linear combination method (Equation (9)). The eigenvalues, variance percentages, cumulative variance percentages, and calculated CRITIC weights for the extracted principal components (PC I–IV) are summarized in Table 2.

(6) $$C_{k}=\sum_{l=1}^{m}(1\;-\;|r_{kl}\left|\right)$$

(7) $$I_{k}=\sigma_{k}\times C_{k}$$

(8) $$w_{k}=\frac{I_{k}}{\sum_{k=1}^{m}I_{k}}$$

(9) $$S_{i}=\sum_{k=1}^{m}w_{k}\times PC_{ik}^{norm}$$

where C*_k_* is the information conflict between the k-th principal component and other components; I*_k_* denotes the comprehensive information capacity of the k-th component; |r*_kl_*| is the absolute correlation coefficient between the k-th and l-th components; *σ_k_* represents the standard deviation of the k-th component; w*_k_* is the CRITIC weight for the k-th component (satisfying $\sum_{k=1}^{m}w_{k}=1$); and S*_i_* signifies the comprehensive high photosynthetic efficiency score of the i-th sample, with a higher score indicating a superior photosynthetic level.

### 4.3. UAV Multimodal Data Acquisition and Processing

#### 4.3.1. Image Acquisition and Processing

A DJI Mavic 3M UAV, equipped with a high-resolution CMOS visible (RGB) camera and a four-channel multispectral sensor-covering green (560 nm), red (650 nm), red-edge (730 nm), and near-infrared (860 nm) bands-was utilized for multimodal remote sensing data acquisition (Figure 9b). UAV flights were synchronized with ground-based field measurements and executed at an altitude of 30 m and a speed of 2 m/s, with forward and side overlaps set at 80% and 70%, respectively. Flight routes were automated via DJI Pilot 2 (v10.1.5.10) using an equal-time interval triggering mode. To ensure geometric and radiometric consistency, 1 m × 1 m ground control points (GCPs) were deployed around the experimental area, and their centimeter-level coordinates were acquired using a real-time kinematic (RTK) base station. Additionally, images of a standard radiometric calibration panel were captured prior to takeoff to eliminate sensor and ambient lighting distortions.

The acquired raw RGB and multispectral (MS) images were imported into Pix4Dmapper software. By incorporating ground control points (GCPs) and manually marking tie points, a bundle block adjustment (BBA) was performed to generate densified point clouds and textured meshes. Following radiometric calibration and geometric correction, high-resolution RGB and MS orthomosaics were generated. These orthomosaics were then imported into ENVI 5.6 software, where threshold segmentation based on the Normalized Difference Vegetation Index (NDVI) was applied to distinguish the crop canopy from the soil background [8]. Statistical tools were utilized for comparative analysis to extract binary vector masks, which were subsequently used to crop the regions of interest (ROIs), effectively eliminating soil background interference.

#### 4.3.2. Extraction of Vegetation Indices

To investigate the spectral response characteristics of the CHPPI across different wheat varieties, 12 RGBVIs and 12 MSVIs closely associated with wheat growth conditions were selected for analysis. The specific mathematical formulas for these vegetation indices are detailed in Table 3.

#### 4.3.3. Acquisition of Texture Features

As a fundamental visual characteristic of images, texture manifests as combinations of similar patterns with varying intensities. In remote sensing image analysis, spatial texture serves as a critical property distinct from spectral characteristics. In this study, texture features were extracted based on the Gray Level Co-occurrence Matrix (GLCM) method [77], which quantitatively describes the spatial structure of textures by calculating the joint gray-level distribution between pixel pairs at specific directions and distances [78].

Using the “Co-occurrence Measures” tool in ENVI 5.6 software, GLCMs were constructed separately for the four multispectral bands. For each band image, eight categories of texture metrics were systematically extracted: mean (MEA), variance (VAR), homogeneity (HOM), contrast (CON), dissimilarity (DIS), entropy (ENT), second moment (SEC), and correlation (COR). These features characterize the texture in terms of image brightness, local variation, uniformity, structural contrast, degree of difference, informational complexity, distribution uniformity, and linear correlation, respectively. To evaluate the sensitivity of CHPPI estimation to spatial texture scales, a comparative analysis was conducted across multiple spatial window sizes (3 × 3, 5 × 5, 7 × 7, and 9 × 9) (Table S1). Based on these results, a 5 × 5 moving window with a step size of 1 pixel was selected to compute the GLCM textures, balancing feature stability with the preservation of spatial details while ensuring spatial continuity in the feature maps. Additionally, directional averaging across four orientations (0°, 45°, 90°, and 135°) was applied to achieve rotational invariance and eliminate potential bias caused by crop row directions.

### 4.4. Estimation Model Construction

#### 4.4.1. Feature Selection Methods

To eliminate redundancy from the multimodal dataset containing RGBVIs, MSVIs, and TFs, four methods were compared to screen for the optimal variable subset. The Pearson correlation coefficient (PCC) method computes the correlation coefficients between independent and dependent variables combined with significance tests (*p* < 0.01; variables with *p* ≥ 0.01 were excluded) to identify sensitive features directly contributing to the target variable [79]. The variable importance in projection (VIP) method scores explanatory variables based on their weight vectors in a partial least squares regression (PLSR) model where the optimal number of latent components was automatically optimized [80]; variables with a VIP value greater than 1.0 (VIP > 1.0) were selected as predictors, offering a distinct advantage when handling datasets with substantial multi-collinearity. To address severe collinearity among features, the successive projections algorithm (SPA) utilizes vector space projection analysis to continuously minimize linear redundancy during screening, evaluating candidate subsets via 5-fold cross-validation coupled with linear regression to automatically select the feature combination corresponding to the minimum cross-validation RMSE (CV-RMSE) to extract the least redundant feature combination with clear physical significance while maintaining informational integrity [81]. Lastly, the uninformative variable elimination (UVE) method introduces artificial random noise (standard normal distribution N(0, 1), 100 Monte Carlo PLS iterations with 5 components) into the original variables and performs stability analysis based on an adaptive threshold (defined as the mean plus one standard deviation of the noise reliability indices) to eliminate features with negligible or no contribution to the predictions, thereby enhancing model robustness [41].

#### 4.4.2. Machine Learning Algorithms

Five representative machine learning algorithms were selected to handle the non-linear relationships within the multimodal remote sensing data and construct quantitative estimation models for wheat CHPPI. As a non-parametric method, the K-nearest neighbors (KNN) algorithm predicts outcomes via a locally weighted average of the k training samples closest in Euclidean distance to the target sample within the feature space [82]. The decision tree (DT) recursively splits sample features to construct a tree-like decision logic, offering high interpretability and robust resistance to outliers [83].Support vector regression (SVR) maps input features into a high-dimensional space via kernel functions, seeking a globally optimal hyperplane to address complex non-linear regression tasks [84]. Within ensemble learning, random forest (RF) leverages the Bagging strategy to construct multiple decision trees in parallel, averaging the outputs of weak learners to effectively suppress overfitting [85]. Meanwhile, Extreme Gradient Boosting (XGBoost), an advanced Boosting framework, optimizes the loss function using a second-order Taylor expansion and incorporates regularization to control model complexity, demonstrating excellent performance in quantitative crop phenotype estimation [86]. In addition, feature standardization was applied to both the single feature subsets (MSVI, RGBVI, and TF) and their combination, Comprehensive Multispectral-Visible-Texture Features (CMVTF), prior to model training to eliminate scale and dimensional discrepancies across feature variables. To ensure optimal performance, a 5-fold cross-validation combined with grid search was uniformly employed to optimize the core hyperparameters of each algorithm. The tuning search spaces and final optimal hyperparameter configurations for all five evaluated models (XGBoost, RF, SVR, DT, and KNN) are detailed in Table S2.

#### 4.4.3. Model Evaluation Metrics

**Figure 10:**
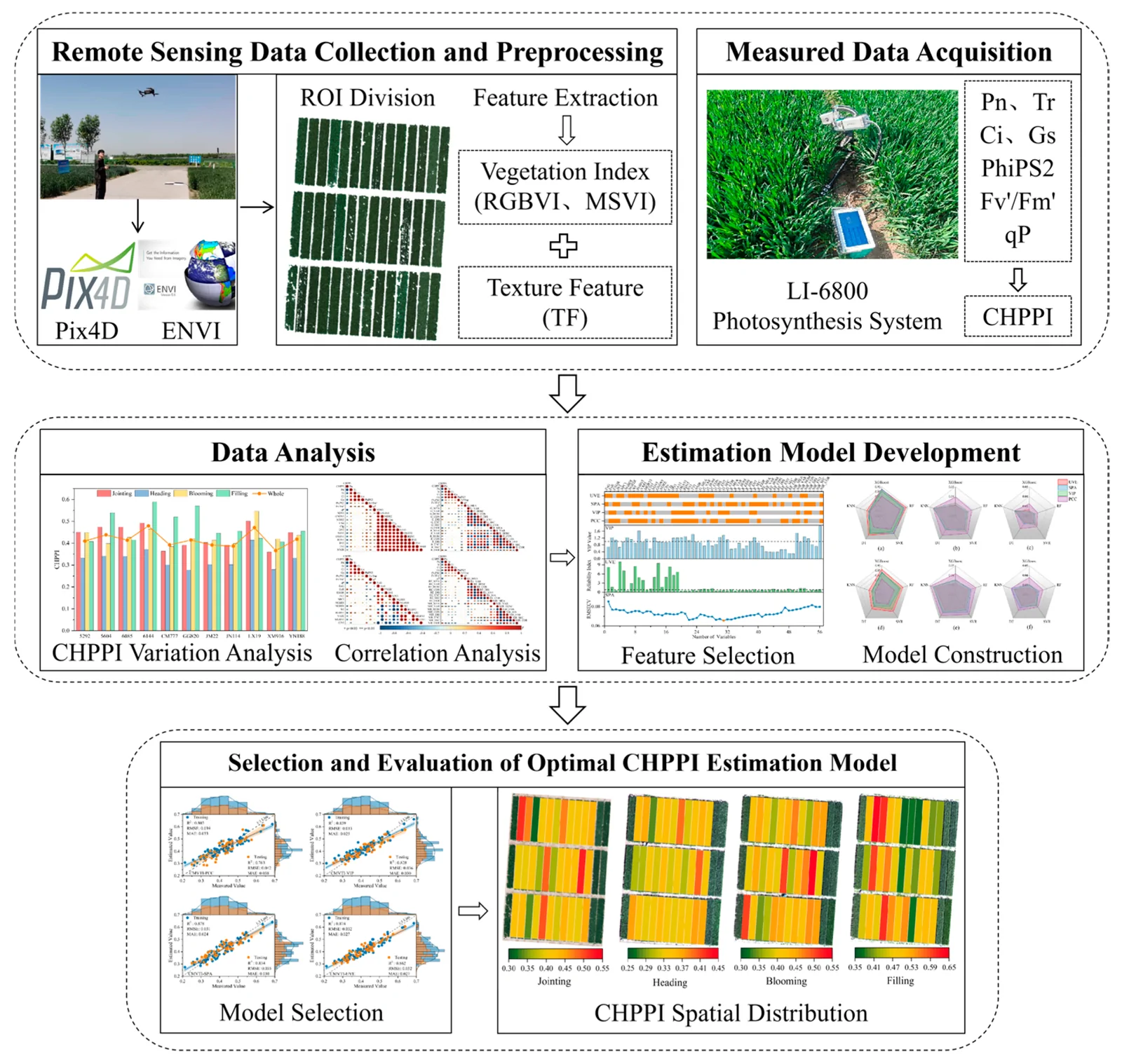
Flowchart of experimental research.

In this study, the Kennard-Stone (KS) algorithm was applied to randomly partition the samples at the sample level [87], with two-thirds allocated as the training dataset for model calibration and the remaining one-third designated as the testing dataset for validation. The detailed workflow of model construction and evaluation based on UAV imagery is shown in Figure 10. To assess the performance and reliability of the estimation models, standard evaluation metrics were adopted following the general methodology of machine learning evaluation [88]. Specifically, the coefficient of determination (R^2^), root mean square error (RMSE), and mean absolute error (MAE) were used to evaluate model accuracy. These metrics are mathematically defined as follows:

(10) $$R^{2}=1-\frac{\sum_{i=1}^{n}{\left(y_{i}-{\hat{y}}_{i}\right)}^{2}}{\sum_{i=1}^{n}{\left(y_{i}-\bar{y}\right)}^{2}}$$

(11) $$RMSE=\sqrt{\frac{1}{n}\sum_{i=1}^{n}{\left(y_{i}-{\hat{y}}_{i}\right)}^{2}}$$

(12) $$MAE=\frac{1}{n}\sum_{i=1}^{n}\left|y_{i}-{\hat{y}}_{i}\right|$$

where $y_{i}$ and ${\hat{y}}_{i}$ represent the measured and estimated values of the *i*-th sample, respectively; $\bar{y}$ denotes the mean of the measured values; and *n* is the total number of samples. Higher R^2^ values, combined with lower RMSE and MAE values, indicate greater accuracy of the CHPPI estimation model.

## 5. Conclusions

This study developed a wheat cultivar screening method based on UAV multimodal remote sensing data. By integrating multiple photosynthetic phenotypic parameters via the PCA-CRITIC method, a Comprehensive High Photosynthetic-Efficiency Phenotypic Index (CHPPI) was successfully constructed. The Comprehensive Multispectral-Visible-Texture Features (CMVTF) were coupled with optimal feature selection and machine learning algorithms to achieve precise CHPPI estimation. The primary conclusions are as follows:

(1) The CHPPI effectively captured cultivar-specific variations in photosynthetic productivity, with cultivars SH06144 and Liangxing19 consistently maintaining high photosynthetic efficiency throughout the entire growth period.
(2) The CMVTF outperformed single feature categories in estimation performance. Compared with PCC, VIP, and SPA, the UVE was most effective in eliminating collinearity and noise, and its selected feature subset significantly enhanced model robustness.
(3) The ensemble algorithms (XGBoost and RF) outperformed traditional models. The CMVTF-UVE-XGBoost model achieved the optimal accuracy (R^2^ = 0.862, RMSE = 0.032, and MAE = 0.027). Furthermore, the spatial distribution of estimated CHPPI was highly consistent with measured values, validating its operational reliability.

In summary, the developed CMVTF-UVE-XGBoost framework can effectively and accurately evaluate wheat photosynthetic efficiency, thereby providing robust technical support for the large-scale screening of high photosynthetic efficiency breeding materials.

## Figures and Tables

**Table 1:** CHPPI estimation results based on different feature variables.

Type	Model	Training Set			Testing Set		
		R^2^	RMSE	MAE	R^2^	RMSE	MAE
MSVI	XGBoost	0.764	0.043	0.036	0.674	0.049	0.041
	RF	0.774	0.042	0.032	0.654	0.050	0.042
	SVR	0.657	0.052	0.037	0.647	0.051	0.040
	DT	0.686	0.050	0.039	0.634	0.052	0.043
	KNN	0.6845	0.0499	0.0374	0.6181	0.0529	0.0435
RGBVI	XGBoost	0.659	0.052	0.040	0.626	0.052	0.045
	RF	0.673	0.051	0.038	0.603	0.054	0.046
	SVR	0.624	0.055	0.041	0.618	0.053	0.043
	DT	0.679	0.050	0.042	0.586	0.055	0.043
	KNN	0.574	0.058	0.046	0.554	0.057	0.046
TF	XGBoost	0.745	0.045	0.036	0.650	0.051	0.043
	RF	0.733	0.046	0.039	0.663	0.050	0.043
	SVR	0.661	0.052	0.044	0.647	0.051	0.042
	DT	0.702	0.049	0.041	0.648	0.051	0.042
	KNN	0.662	0.052	0.043	0.606	0.054	0.043
CMVTF	XGBoost	0.818	0.038	0.030	0.725	0.045	0.038
	RF	0.791	0.041	0.032	0.703	0.047	0.038
	SVR	0.715	0.047	0.039	0.694	0.047	0.039
	DT	0.755	0.044	0.035	0.665	0.050	0.042
	KNN	0.665	0.052	0.040	0.643	0.051	0.043

**Table 2:** Principal component characteristics and CRITIC weights.

	Principal Component			
	Ι	II	III	IV
Eigenvalues	3.55	1.623	0.822	0.449
Variance percentages (%)	50.33	23	11.65	6.36
Cumulative variances percentages (%)	50.33	73.33	84.98	91.34
CRITIC weights	0.29	0.26	0.20	0.25

**Table 3:** Vegetation indices calculated based on the RGB and multispectral images of UAV.

Type	Vegetation Index	Formulation	References
RGBVIs	EXR	1.4r − g	[53]
	EXG	2g − r − b	[54]
	EXGR	3g − 2.4r − b	[55]
	INT	(r + g + b)/3	[56]
	NPCI	(r − b)/(r + b)	[57]
	NGBDI	(g − b)/(g + b)	[58]
	NGRDI	(g − r)/(g + r)	[59]
	WI	(g − b)/(r − g)	[60]
	RGBVI	(g^2^ − r × b)/(g^2^ + r × b)	[61]
	VARI	(g − r)/(g + r-b)	[62]
	MGRVI	(g^2^ − r^2^)/(g^2^ + r^2^)	[63]
	CIVI	0.44r − 0.88g + 0.39b + 18.7875	[64]
MSVIs	NDVI	(NIR − R)/(NIR + R)	[65]
	GNDVI	(NIR − G)/(NIR + G)	[66]
	NDRE	(NIR − RE)/(NIR + RE)	[67]
	CIre	NIR/RE − 1	[68]
	CIg	NIR/G − 1	[69]
	DVI	NIR − R	[70]
	SAVI	1.5 × (NIR − R)/(NIR + R + 0.5)	[71]
	OSAVI	1.16 × (NIR − R)/(NIR + R + 0.16)	[72]
	RDVI	(NIR − R)/sqrt(NIR + R)	[73]
	RVI	NIR/R	[74]
	TVI	60 × (NIR − G) − 100 × (R − G)	[75]
	NNIR	NIR/(NIR + RE + G)	[76]

Note: r, g, and b represent the blue, green, and red bands of the visible (RGB) camera, respectively; G, R, RE, and NIR denote the green, red, red-edge, and near-infrared bands of the multispectral sensor, respectively.

## Data Availability

The original contributions presented in this study are included in the article/Supplementary Material. Further inquiries can be directed to the corresponding author.

## Supplementary Materials

The following supporting information can be downloaded at https://www.mdpi.com/article/10.3390/plants15172674/s1: Table S1: CHPPI estimation performance using XGBoost based on GLCM texture features across spatial window sizes; Table S2: Hyperparameter search spaces and optimal values for machine learning algorithms.

## Abbreviations

The following abbreviations are used in this manuscript:

UAV	Unmanned aerial vehicle
PCA	Principal component analysis
CRITIC	CRiteria Importance Through Intercriteria Correlation
CHPPI	Comprehensive High Photosynthetic-Efficiency Phenotypic Index
MSVI	Multispectral vegetation indices
RGBVI	RGB vegetation indices
TF	Texture features
CMVTF	Comprehensive Multispectral-Visible-Texture Features
PCC	Pearson correlation coefficient
VIP	Variable importance in projection
SPA	Successive projections algorithm
UVE	Uninformative variable elimination
KNN	K-nearest neighbors
DT	Decision tree
SVR	Support vector regression
RF	Random forest
XGBoost	Extreme gradient boosting
Pn	Net photosynthetic rate
Tr	Transpiration rate
Ci	Intercellular CO_2_ concentration
Gs	Stomatal conductance
PhiPS2	Photochemical quantum efficiency
Fv’/Fm’	PSII reaction center excitation energy capture efficiency
qP	Photochemical quenching coefficient
