# Supplementary material for: Estimation of the Comprehensive High Photosynthetic-Efficiency Phenotypic Index in Winter Wheat Based on UAV Multimodal Remote Sensing Data
Source: Plants (Basel). 2026 Aug 31;15(17):2674. doi: 10.3390/plants15172674 (PMC13567199; doi:10.3390/plants15172674)
Supplement: Supplementary file 1 [file plants-15-02674-s001.zip › plants-4465567-supplementary.pdf]

Table S1 CHPPI estimation performance using XGBoost based on GLCM texture features across spatial window sizes

**window sizes**	**Training Set**			**Testing Set**		
	R^2	RMSE	MAE	R^2	RMSE	MAE
3 × 3	0.740	0.045	0.040	0.629	0.052	0.045
5 × 5	0.745	0.045	0.036	0.650	0.051	0.043
7 × 7	0.746	0.045	0.039	0.620	0.053	0.047
9 × 9	0.710	0.048	0.039	0.602	0.054	0.048

Table S2 Hyperparameter search spaces and optimal values for machine learning algorithms.

**Algorithm**	**Key Hyperparameters**	**Search Space / Tuning Range**	**Optimal Value**
KNN	n_neighbors	[1, 15] (step = 1)	5
	weights	['uniform', 'distance']	'distance'
	metric	['euclidean', 'manhattan']	'euclidean'
DT	max_depth	[3, 20] (step = 1)	7
	min_samples_split	[2, 10] (step = 1)	5
	min_samples_leaf	[1, 5] (step = 1)	2
SVR	kernel	['linear', 'rbf', 'poly']	'rbf'
	C	[10-2, 103] (log space)	10
	gamma	[10-3, 101] / ['scale', 'auto']	'scale'
	epsilon	[0.01, 0.5] (step = 0.01)	0.1
RF	n_estimators	[50, 500] (step = 50)	200
	max_depth	[2, 20] (step = 1)	10
	max_features	['sqrt', 'log2', None]	'sqrt'
	min_samples_split	[2, 10] (step = 1)	2
XGBoost	n_estimators	[50, 500] (step = 50)	150
	learning_rate	[0.01, 0.3] (step = 0.01)	0.1
	max_depth	[3, 10] (step = 1)	5
	subsample	[0.5, 1.0] (step = 0.1)	0.8
	colsample_bytree	[0.5, 1.0] (step = 0.1)	0.8
